# Investigating Eye Movement and Postural Stability Relationships Using Mobile Eye-Tracking and Posturography: A Cross-Sectional Study

**DOI:** 10.3390/bioengineering11080742

**Published:** 2024-07-23

**Authors:** Seo-Yoon Park, Tae-Woo Kang, Dong-Kyun Koo

**Affiliations:** 1 Department of Physical Therapy, College of Health and Welfare, Woosuk University, 443 Samnye-ro, Samnye-eup, Wanju-gun 55338, Republic of Korea; pgy0614@hanmail.net (S.-Y.P.); ktwkd@hanmail.net (T.-W.K.); 2 HiVE Center, University-Industry Foundation, Wonkwang Health Science University, 514, Iksan-daero, Iksan-si 54538, Republic of Korea

**Keywords:** mobile eye-tracking, posturography, sensory integration

## Abstract

Vision and eye movements play a crucial role in maintaining postural stability. This study investigated the relationship between eye movements and postural control in healthy adults using mobile eye-tracking technology and posturography. Forty healthy participants underwent assessments of eye movements using a mobile eye-tracking system and postural stability using Tetrax posturography under various sensory conditions. Pearson correlation coefficients were computed to examine associations between eye movement parameters and postural control indices. Significant correlations were found between eye movement parameters and postural stability indices. Faster and more consistent horizontal eye movements were associated with better postural stability (r = −0.63, *p* < 0.05). Eye movement speed variability positively correlated with weight distribution indices under normal eyes open (r = 0.65, *p* < 0.05) and closed (r = 0.59, *p* < 0.05) conditions. Coordination of horizontal and vertical eye movements positively correlated with postural control (r = 0.69, *p* < 0.01). Negative correlations were observed between eye movement coordination and Fourier indices in various frequency bands (*p* < 0.05) and the stability index under different head positions (*p* < 0.05). The findings provide insights into sensory integration mechanisms underlying balance maintenance and highlight the importance of integrated sensory processing in postural stability. Eye movement assessments have potential applications in balance evaluation and fall risk prediction.

## 1. Introduction

Vision and eye movements play a pivotal role in the perception of and interaction with the external environment, enabling crucial tasks such as recognition, localization, and proprioception [1,2]. These functions are essential for perceiving the world around us and maintaining an upright posture and balance [3]. Vision facilitates the detection of self-motion relative to the environment, which is critical for postural stabilization [4,5].

Traditionally, postural stabilization has been linked to changes in the retinal image [6]. The central nervous system utilizes motion-induced optic flow patterns on the retina to estimate body position and make appropriate postural adjustments [7,8]. Previous studies have demonstrated that body sway is linked to stimulus motion, indicating the central nervous system’s ability to interpret external motion as self-motion and adjust body orientation accordingly [9].

Eye movements, including smooth pursuit and saccadic movements, have been shown to have a significant influence on postural control [10,11], suggesting a complex relationship between gaze behavior and postural modulation [12]. Advancements in mobile eye-tracking technology have revolutionized this field, providing a practical and accessible means to measure and analyze eye movements in everyday settings.

Recent studies utilizing mobile eye-tracking systems have provided valuable insights into the relationship between eye movements and postural control. For instance, researchers have investigated gaze behavior during natural locomotion [13], explored visual exploration strategies in complex environments [14], examined gaze behavior changes in response to different walking speeds [15], and combined eye-tracking with posturography to study visual search strategies and postural sway [16]. These studies have consistently demonstrated links between eye movement patterns, visual strategies, and postural stability, highlighting the potential for eye movement assessment and training in balance-related applications.

The current study aimed to investigate the relationship between eye movements and postural stability in healthy adults using mobile eye-tracking technology and posturography. By employing mobile devices, we sought to provide a more comprehensive understanding of the integration of vision and posture in daily activities. The findings of this research have important implications for clinical practice and further scientific inquiries in the fields of vision and motor control.

## 2. Materials and Methods

### 2.1. Subjects

This study included 40 healthy young adults (age range: 18–35 years) who were recruited through advertisements at a university campus. The sample size was determined based on a power analysis using G*Power software (version 3.1.9.7). Given the exploratory nature of our study investigating the relationship between eye movements and postural stability using mobile eye-tracking and posturography, we aimed to detect medium effect sizes. Using parameters for a correlation analysis (two-tailed test, α error probability of 0.05, power (1−β error probability) of 0.8, and a medium effect size of r = 0.4), the analysis yielded a minimum sample size of 37 participants. We increased our sample to 40 to account for potential dropouts and to improve the reliability of our findings. The inclusion criteria were no history of musculoskeletal, neurological, or psychiatric disorders, and normal or corrected-to-normal vision. Participants with vestibular disorders, uncorrected visual impairments, or any condition that could affect balance or eye movements were excluded. All participants provided written informed consent before participating in this study, which was approved by the Institutional Review Board of Dankook University (DKU 2021-03-069) and conducted in accordance with the Declaration of Helsinki.

### 2.2. Measurements

#### 2.2.1. Eye-Tracker

**Figure 1:**
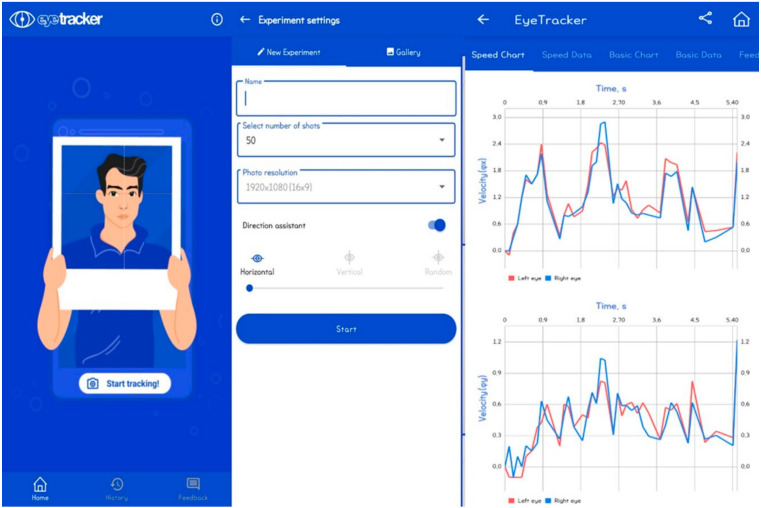
EyeTracker mobile eye-tracking device and task.

A portable eye-tracking system (EyeTracker, version 1.9.4, BVG Software Group LLC, South San Francisco, CA, USA) was used to continuously track binocular eye movements at a sampling rate of 20 Hz (Figure 1). The device consisted of a mobile tablet with a built-in camera and infrared sensors to detect eye positions. The tablet was mounted on a tripod, and participants were instructed to keep their head as still as possible during the tests. Participants were asked to stand during the eye-tracking assessment to mimic natural viewing conditions and maintain consistency with the subsequent posturography assessment. To ensure minimal head movement, participants were explicitly instructed to maintain a stable head position throughout the eye-tracking assessment. While no additional physical restraints were used, the importance of head stability was emphasized to the participants before and during the tests.

The eye-tracking task involved tracking a red circular marker (0.3 cm diameter) displayed on the tablet screen. The marker moved horizontally or vertically at a constant speed of 10 degrees per second for 15 s in each direction. Participants completed three trials for each direction (horizontal and vertical), with a 10 s rest between trials. A dark screen was displayed for 10 s before each trial to allow for eye adjustment.

Raw eye-tracking data were processed using the EyeTracker software’s network refinement feature, which automatically filtered out low-quality frames affected by blinks, incorrect initial frames, or unrecognized pupils. The 50 most accurate consecutive frames (2.5 s) from each trial were selected for analysis. The following parameters were calculated for both eyes in horizontal (HOR) and vertical (VER) conditions:

- Average speed (degrees/second): HorSpeed, VerSpeed;
- Coefficient of variation (%): HorCV, VerCV;
- Pearson correlation coefficients between left and right eye speeds:
  – HOR condition: HorCorr, HorVerCorr;
  – VER condition: VerHorCorr, VerCorr.

#### 2.2.2. Tetrax Posturography

**Figure 2:**
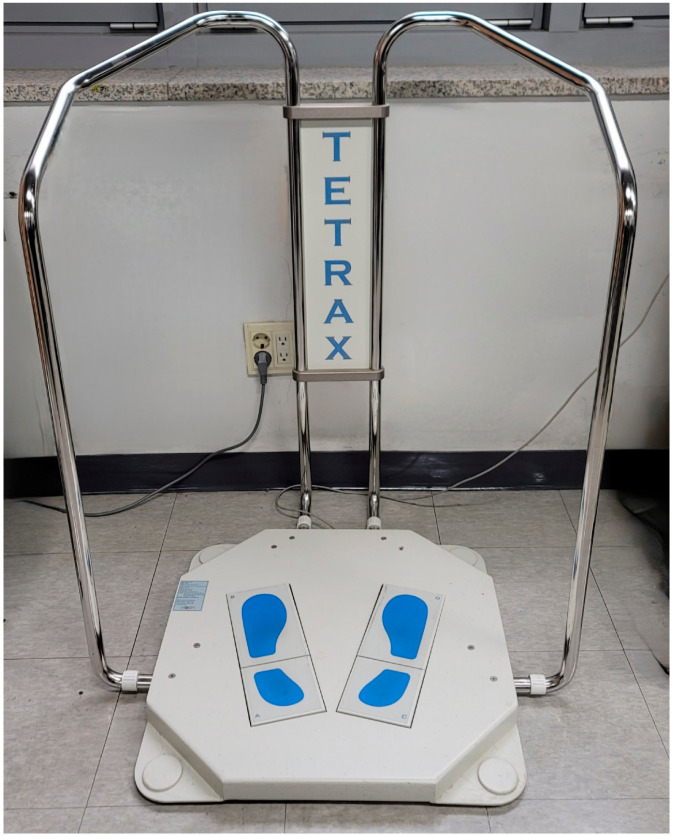
Tetra-ataxiometric posturography system setup.

Postural stability was assessed using the Tetrax posturography system (Sunlight Medical Ltd., Ramat Gan, Israel), which consists of four rectangular force plates (A, B, C, D) arranged in a diamond configuration (Figure 2). The anterior plates (B and D) measure 12 cm × 19 cm, while the posterior plates (A and C) are 12 cm × 12 cm. Pressure sensors beneath each plate detect weight distribution and postural sway, with the data transmitted to a computer for analysis using Tetrax software.

Participants stood barefoot on the force plates with their arms at their sides and eyes facing forward. They were instructed to minimize movement during the tests. Eight 32 s test conditions were performed in a randomized order:

- Normal eyes open (NO): This condition serves as a baseline, representing typical everyday standing posture with full sensory input.
- Normal eyes closed (NC): By removing visual input, this condition assesses the reliance on vestibular and proprioceptive systems for balance maintenance, simulating situations like standing in a dark room.
- Head right (HR) and head left (HL): These conditions, with eyes closed and head turned at least 45° to the right or left, evaluate vestibular function and the ability to maintain balance with altered head orientation, mimicking everyday activities like looking sideways while standing.
- Head back (HB): With eyes closed and head tilted backward at least 30°, this condition assesses balance control during cervical extension, which is relevant for activities like looking up at high shelves or ceiling work.
- Head forward (HF): Eyes closed with head bent forward at least 30°, this condition evaluates balance during cervical flexion, simulating tasks such as reading or looking at a smartphone while standing.
- Pillow with eyes open (PO) and pillow with eyes closed (PC): These conditions, performed on a foam pillow, challenge proprioceptive input by creating an unstable surface. They assess the integration of visual (PO) or vestibular (PC) information with altered somatosensory input, simulating standing on uneven or soft surfaces like carpet or sand.

These diverse conditions were chosen to comprehensively assess postural control under various sensory challenges that reflect real-world situations. They allow for the evaluation of how individuals integrate and prioritize different sensory inputs (visual, vestibular, and proprioceptive) to maintain balance, providing insights into potential deficits or compensatory strategies in postural control.

The Tetrax software calculated several parameters:

- Stability index (ST): a measure of overall postural stability, with higher values indicating greater instability. This index is clinically relevant for assessing fall risk and overall balance performance.
- Fourier indices (F1–F8): a regression analysis of postural sway intensity at different frequency bands, with each band associated with specific sensory or neurological functions:
- F1 (<0.1 Hz): visual–vestibular regulation;
- F2–F4 (0.1–0.5 Hz): vestibular disorders;
- F5–F6 (0.5–1 Hz): somatosensory disorders in the lower limbs and spine;
- F7–F8 (>1 Hz): central nervous system disorders. These indices provide detailed information about the underlying mechanisms of postural control and can help identify specific areas of dysfunction.
- Weight Distribution Index (WDI): the percentage of body weight on each force plate, with deviations from 25% per plate indicating postural asymmetry. This index is particularly relevant for assessing balance in individuals with unilateral impairments or injuries.
- Synchronization Index (SI): the similarity of postural sway patterns between pairs of force plates, with positive values indicating in-phase synchronization and negative values indicating anti-phase synchronization. This index provides insights into the coordination of postural adjustments and can be useful in identifying subtle balance disorders.

By analyzing these parameters across the various test conditions, we can gain a comprehensive understanding of an individual’s postural control strategies and potential areas of deficit, which can inform both clinical assessment and rehabilitation strategies.

#### 2.2.3. Experimental Procedure

This study was conducted in a controlled laboratory environment with minimal visual and auditory distractions. Upon arrival, participants were informed about the study’s purpose and procedures, and written informed consent was obtained. Participants then completed a demographic questionnaire.

The experimental session commenced with the eye-tracking assessment. The mobile eye-tracking device was calibrated for each participant using a 9-point calibration procedure. Participants were instructed to maintain a stable head position and track a circular marker with their eyes as it moved horizontally or vertically on the screen. The marker moved at a constant velocity for 15 s in each direction, with a 5 s static display between directions. Participants completed three trials for each direction (horizontal and vertical), with a 30 s inter-trial interval. A 1 min break was provided upon completion of the eye-tracking assessment.

Subsequently, participants proceeded to the posturography assessment. They were instructed to remove their footwear and stand barefoot on the Tetrax force plates, with their feet positioned according to the manufacturer’s guidelines. The force plates were calibrated for each participant’s body weight. Participants were instructed to maintain an upright stance with their arms at their sides and their gaze directed forward. They were also instructed to refrain from talking or making extraneous movements during the testing.

The eight posturography test conditions were administered in a randomized order, with each condition lasting for 32 s. The conditions involved varying combinations of visual input (eyes open or closed), head orientation (forward, right, left, back, or bent forward), and surface type (firm or foam). For the eyes closed conditions, participants were instructed to close their eyes gently without excessive squeezing. For the head orientation conditions, participants were instructed to maintain the specified head position throughout the trial. In the foam surface conditions, participants stood on a high-density foam pillow (dimensions: 12 cm × 31 cm × 5 cm, covering the entire surface area of the force plates and elevating the feet 10 cm from the ground) placed atop the force plates. The anterior force plates (B and D) measured 12 cm × 19 cm, while the posterior force plates (A and C) measured 12 cm × 12 cm, resulting in a total length of 31 cm for the combined force plate surface. A 30 s rest period was provided between each condition, during which participants could open their eyes and relax their posture.

The total duration of the experimental session, including both eye-tracking and posturography assessments, was approximately 30 min. Participants were encouraged to take additional rest breaks as needed to minimize fatigue. The order of the eye-tracking and posturography assessments was counterbalanced across participants to control for potential order effects. Upon completion of both assessments, participants were debriefed about the study and provided with an opportunity to ask questions. They were thanked for their participation and provided with contact information for the research team for any follow-up concerns or inquiries.

#### 2.2.4. Statistical Analysis

Data were analyzed using SPSS software (version 26.0, IBM Corp., Armonk, NY, USA). Descriptive statistics (mean ± standard deviation) were calculated for all variables. Pearson correlation coefficients were used to examine the relationships between eye-tracking parameters and posturography indices, with *p*-values < 0.05 considered statistically significant. Scatter plots were generated to visualize the correlations between key variables.

## 3. Results

### 3.1. Participant Characteristics

This study included 40 healthy participants (18 males, 22 females) with a mean age of 26.95 ± 4.23 years, a mean height of 169.20 ± 6.31 cm, and a mean weight of 64.20 ± 8.44 kg.

### 3.2. Correlations between Eye Movement Parameters and Postural Stability Indices

**Figure 3:**
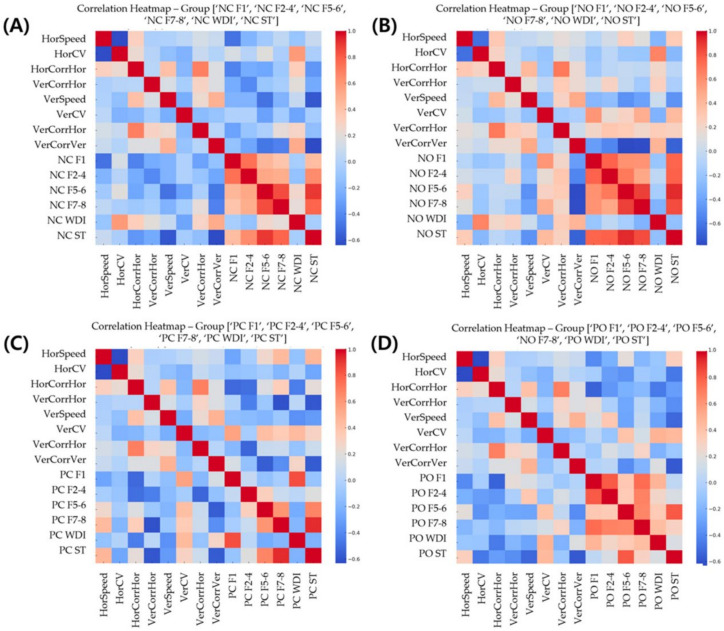
Correlation Heatmaps of Eye Movement Parameters and Postural Stability Indice. Heatmaps illustrating correlations between eye movement parameters (average speed, coefficient of variation [CV], and left–right correlations for horizontal [HOR] and vertical [VER] directions) and postural stability indices (Fourier indices [F1, F2–F4, F5–F6, F7–F8], Weight Distribution Index [WDI], and stability index [ST]) under four conditions: (**A**) normal eyes closed (NC), (**B**) normal eyes open (NO), (**C**) pillow with eyes closed (PC), and (**D**) pillow with eyes open (PO). Color scale ranges from −0.6 (blue, negative correlation) to 1.0 (red, positive correlation). Intensity of color indicates strength of correlation.

Pearson correlation analysis revealed significant associations between various eye movement parameters and postural stability indices (Table 1, Figure 3). HorSpeed showed a moderate negative correlation with HorCV (r = −0.63, *p* < 0.05). HorCorr exhibited a strong positive correlation with VerHorCorr (r = 0.69, *p* < 0.01).

**Figure 4:**
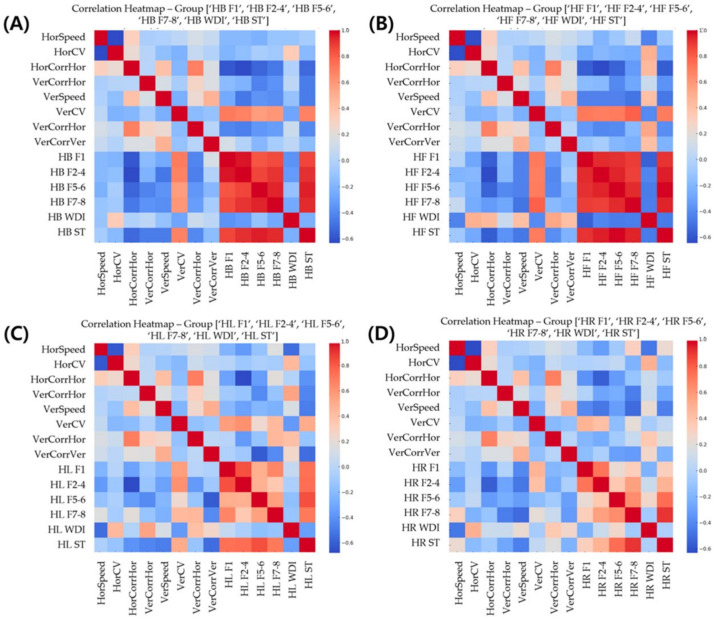
Correlation Heatmaps of Eye Movement Parameters and Postural Stability Indices Under Various Head Positions. Heatmaps illustrating correlations between eye movement parameters and postural stability indices (as defined in Figure 1) under four head position conditions: (**A**) head back (HB), (**B**) head forward (HF), (**C**) head left (HL), and (**D**) head right (HR). Color scale ranges from −0.6 (blue, negative correlation) to 1.0 (red, positive correlation), with color intensity indicating correlation strength. These heatmaps demonstrate significant associations between visual and postural control systems across different head orientations.

HorCorr was negatively correlated with several postural stability indices, including F2–F4 under the HR (r = −0.56, *p* < 0.05) and HL (r = −0.68, *p* < 0.05) conditions, as well as F1, F2–F4, F5–F6, and ST under the HB and HF conditions (*p* < 0.05) (Figure 4).

VerCorr showed negative correlations with F7–F8 and ST under the PC condition (*p* < 0.05). In contrast, VerCV was positively correlated with various postural stability indices, as evident from the moderate to strong correlations observed between VerCV and F1, F2–F4, F5–F6, F7–F8, and ST under the NO, PC, HL, HB, and HF conditions (*p* < 0.05).

Additionally, HorCV was positively correlated with WDI under the NO (r = 0.65, *p* < 0.05) and NC (r = 0.59, *p* < 0.05) conditions (Figure 3).

## 4. Discussion

The present study employed advanced eye-tracking technology and Tetrax posturography to investigate the intricate relationship between eye movements and postural stability in a sample of healthy adults. The findings provide valuable insights into the complex sensory integration mechanisms underlying the maintenance of balance and have important implications for both clinical practice and research in the fields of vision and motor control.

One of the key findings of this study was the negative correlation between the average speed of horizontal eye movements and their variability, suggesting that faster and more consistent eye movements are associated with better postural stability. This relationship may be indicative of an efficient vestibulo-ocular reflex (VOR), which is crucial for maintaining gaze stability during head movements and plays a vital role in spatial orientation and balance [17,18,19,20]. The lower variability in eye movement speed observed in individuals with better postural stability could reflect stable and well-coordinated neural control of ocular motility [21,22]. These findings suggest that assessing eye movement speed variability could potentially serve as a marker for identifying individuals at risk of postural instability and falls, highlighting the clinical relevance of this relationship. While these findings are intriguing, it is important to note that our study was conducted on a sample of healthy young adults, and therefore, the results cannot be directly extrapolated to populations at higher risk of falls, such as older adults or individuals with balance disorders. Future research should investigate whether similar relationships exist in these at-risk populations and explore the potential clinical applications of eye movement assessments in fall risk prediction.

Another notable finding was the positive correlation between eye movement speed variability and weight distribution indices, suggesting that individuals with more variable eye movement speeds may compensate for this inconsistency through adjustments in their postural sway [23,24]. This interpretation is supported by previous studies that have demonstrated the influence of eye movements on postural control [25,26]. For instance, research has shown that saccadic eye movements can enhance postural stability in children, possibly by facilitating the integration of vestibular and somatosensory information [27]. Moreover, studies have found that variability in visual information processing, such as that observed in patients with central vision impairment, can affect postural control and the ability to adapt postural sway in response to changes in visual feedback [28]. While further research is needed to elucidate the underlying neural mechanisms, these findings suggest that the relationship between eye movement variability and weight distribution may reflect a compensatory strategy to maintain balance in the face of visual input inconsistencies.

The strong positive correlation between the coordination of horizontal and vertical eye movements highlighted in this study underscores the importance of efficient eye movement coordination in enhancing three-dimensional spatial perception and postural control [29]. The ability to form a comprehensive visual map of the surroundings through coordinated eye movements is essential for accurate proprioceptive feedback and the maintenance of balance [30,31]. This finding suggests that interventions targeting the improvement of eye movement coordination could potentially enhance postural stability and reduce the risk of falls in various populations, such as older adults or individuals with vestibular disorders [32].

Interestingly, the negative correlations observed between eye movement coordination and various postural control metrics, such as Fourier indices and stability index, indicate that precise visual processing, enabled by highly coordinated eye movements, may contribute to the minimization of postural sway [10,33]. This finding highlights the importance of accurate sensory input in the stabilization mechanisms underlying postural control [25,34] and opens avenues for further research investigating the interactions between different sensory systems in the maintenance of balance [35].

The findings of this study have significant implications for clinical practice and balance intervention design. Incorporating eye movement assessments into balance evaluation protocols could provide a more comprehensive understanding of balance control mechanisms. These insights could inform the development of novel rehabilitation strategies, such as eye movement training programs, potentially enhancing vestibulo-ocular reflex function and postural stability. The observed correlations across different sensory conditions support the development of multi-modal balance training programs. Future research should focus on translating these findings into practical clinical applications, including longitudinal studies on diverse populations to examine the effects of eye movement training on postural stability and explore eye movement parameters as potential predictive markers for fall risk.

While this study provides valuable insights into the relationship between eye movements and postural stability, it is important to acknowledge its limitations. The cross-sectional design and focus on healthy young adults limit the generalizability of our findings to other populations, such as older adults or individuals with visual or vestibular impairments, and preclude the establishment of causal relationships. A significant concern is the use of the EyeTracker system, for which detailed technical specifications and independent validation studies are not readily available, potentially affecting the robustness of our findings. The mobile eye-tracking system, while allowing for more naturalistic assessments, may introduce measurement errors due to head movements or lighting variations. Future research should address these limitations by employing longitudinal designs, including diverse populations, and utilizing thoroughly validated eye-tracking systems. Additionally, more sophisticated statistical approaches, such as multivariate regression models or partial correlations, could account for potential confounding variables and reveal more complex patterns in the data. These enhancements would contribute to a more nuanced understanding of the complex interplay between visual processing and postural control across different age groups, clinical conditions, and real-world environments with varying cognitive loads and visual complexity.

## 5. Conclusions

In conclusion, this study provides a comprehensive understanding of the dynamic interaction between eye movements and postural stability, emphasizing the importance of integrated sensory processing in the maintenance of balance. The findings contribute to the scientific understanding of postural control mechanisms and have the potential to inform the development of targeted interventions for enhancing balance and preventing falls in various populations. Further research is needed to explore the clinical applications of these findings and to investigate the interactions between visual, vestibular, and proprioceptive systems in the context of postural control.

## Figures and Tables

**Table 1:** Correlations between eye movement parameters and postural stability indices.

Variables	HorSpeed	HorCV	HorCorr	VerCorr
HorCV	−0.63 *	-	-	-
VerHorCorr	-	-	0.69 **	-
WDI (NO)	-	0.65 *	-	-
WDI (NC)	-	0.59 *	-	-
F2–F4 (HR)	-	-	−0.56 *	-
F2–F4 (HL)	-	-	−0.68 *	-
F1, F2–F4, F5–F6, ST (HB, HF)	-	-	*	-
F7–F8 ST (PC)	-	-	-	*
F1, F2–F4, F5–F6, F7–F8, ST (NO, PC, HL, HB, HF)	-	-	-	**

HorSpeed: horizontal eye movement speed; HorCV: coefficient of variation for horizontal eye movements; HorCorr: correlation between left and right eye horizontal movements; VerCorr: correlation between left and right eye vertical movements; VerHorCorr: correlation between horizontal and vertical eye movements; WDI: Weight Distribution Index; F1–F8: Fourier indices; ST: stability index; NO: normal eyes open; NC: normal eyes closed; HR: head right; HL: head left; HB: head back; HF: head forward; PC: pillow with eyes closed. * *p* < 0.05, ** *p* < 0.01.

## Data Availability

The datasets generated during and/or analyzed during the current study are not publicly available due to the need to protect the privacy of the participants, but are available from the corresponding author on reasonable request.
